# Robustness to misalignment of low-cost, compact quantitative phase imaging architectures

**DOI:** 10.1364/OSAC.395498

**Published:** 2020-09-17

**Authors:** Catherine R. M. Fitzpatrick, Abby Wilson, Travis W. Sawyer, Peter J. Christopher, Timothy D. Wilkinson, Sarah E. Bohndiek, George S. D. Gordon

**Affiliations:** 1 Department of Engineering, University of Cambridge, 9 JJ Thomson Avenue, Cambridge, CB3 0FA, UK; 2 Department of Physics, Cavendish Laboratory, JJ Thomson Avenue, Cambridge, CB3 0HE, UK; 3 Cancer Research UK Cambridge Institute, Li Ka Shing Centre, Robinson Way, Cambridge, CB2 0RE, UK; 4 Department of Electrical and Electronic Engineering, The University of Nottingham, University Park, Nottingham, NG7 2RD, UK

## Abstract

Non-interferometric approaches to quantitative phase imaging could enable its application in low-cost, miniaturised settings such as capsule endoscopy. We present two possible architectures and both analyse and mitigate the effect of sensor misalignment on phase imaging performance. This is a crucial step towards determining the feasibility of implementing phase imaging in a capsule device. First, we investigate a design based on a folded 4f correlator, both in simulation and experimentally. We demonstrate a novel technique for identifying and compensating for axial misalignment and explore the limits of the approach. Next, we explore the implications of axial and transverse misalignment, and of manufacturing variations on the performance of a phase plate-based architecture, identifying a clear trade-off between phase plate resolution and algorithm convergence time. We conclude that while the phase plate architecture is more robust to misalignment, both architectures merit further development with the goal of realising a low-cost, compact system for applying phase imaging in capsule endoscopy.

## 1. Introduction

Quantitative phase imaging is becoming widely used in microscopy to elucidate previously unresolvable features based on their scattering behaviour and refractive index contrast, for example structural tissue changes indicative of diseases such as cancer [1]. Bringing such measurements into an endoscopic setting could aid clinical decision making by enhancing contrast for early stage disease in vivo [2–4]. A range of diagnostic approaches are currently being developed for capsule endoscopes [5]; this work provides a preliminary look at some key considerations for bringing phase imaging into this setting.

Typical phase imaging systems are based on interferometric techniques, which have strict requirements for alignment and stability and require expensive instrumentation [6,7]. Conversely, non-interferometric approaches exhibit improved stability because they do not require a reference beam [6,8] and have significantly lower optical coherence requirements [9]. Approaches to non-interferometric phase imaging range from using arrays of illumination LEDs to phase-contrast illumination [9], Fourier ptychography using sequentially accessed LED arrays [10] to vertical scanning mechanisms that produce stacks of defocussed intensity images [11]. For implementation in a capsule endoscope, it is reasonable to start with non-interferometric approaches to reduce hardware complexity and cost while increasing compactness.

Hardware complexity is typically reduced by recording intensity snapshots of propagating light beams with commodity CMOS image sensors [12], and minimising the number of additional components. Intensity images are then processed to extract phase, a process termed *phase retrieval*. A 4f correlator architecture, a configuration of two simple lenses, allows the intensity of the object and its 2D Fourier transform to be recorded, a form of phase diversity [13] from which phase can be recovered using the well-known Gerchberg-Saxton algorithm [14]. There are many other phase-retrieval algorithms that use different sets of recorded intensity information and improve robustness by using prior knowledge of the sample structure [15–17]. Modifications to phase retrieval algorithms, such as adding conjugate gradient steps [18], reducing support, or introducing random phase perturbations [19], can allow phase recovery when experimental parameters are not fully characterised. While this architecture is tied strongly to the focal lengths of the lenses, there is scope to adjust the focal lengths and fold the optical paths to produce a compact implementation.

Replacing one lens with a partially transmissive patterned plate, arguably a small increase in hardware complexity, leads to a different set of phase-retrieval algorithms. The use of a phase plate in place of one lens removes one distance constraint, allowing for more compact architectures, while still recording Fourier domain information which allows the recovery of a wider range of scenes without strong prior assumptions about the sample. These plates may partially block light, e.g. coded apertures [20–22], alter only the phase of light, e.g. wavefront coding [23] and Shack-Hartmann wavefront sensors [24], or a mixture of both, e.g. quadriwave lateral shearing interferometry [25].

It is possible to perform holographic microscopy of transmissive samples without any lenses, relying purely on free-space diffraction [26]. However, this requires a priori assumptions about the sample (e.g. sparse support, such as cells on a transparent background) and the system (known diffraction distance) to converge. With typical source to sample distances on the order of centimetres and sample to sensor distances of approximately 1 mm, lensless techniques are less likely than the phase plate and 4f correlator architectures to be applied to capsule endoscopy.

Here we present and examine two architectures for the implementation of phase imaging based on phase retrieval. The approaches do not require any moving parts and do not require prior knowledge of the sample structure. The architectures are chosen because they are compact and require only relatively low-cost components. The first is derived from a 4f correlator, and the second uses a diffractive phase plate in front of a sensor. We evaluate their potential for low-cost manufacture by examining their tolerance to misalignment, a key consideration for determining whether it is feasible to manufacture such devices at scale for endoscopic applications. We present extensive Fourier optics simulations that explore the impact of axial and transverse sensor misalignment, and implement novel modifications to well-established phase retrieval algorithms in order mitigate the impact of misalignment. We also compare our phase imaging simulations with experimental 4f correlator results produced using off-the-shelf components, demonstrating that the technique can be implemented with low-cost sensors and showing good agreement between experiment and simulation. We discuss cost drivers for the production of the two architectures and propose that they both merit further investigation in the context of developing low-cost, compact phase imaging for capsule endoscopy. We anticipate that our novel analysis of robustness to misalignment, in the context of low-cost manufacturability, will aid design decisions for future capsule endoscopes and other low-cost phase imaging devices.

## 2. Methods

### 2.1 4f architecture

#### 2.1.1 Simulation: phase imaging

**Fig. 1.**
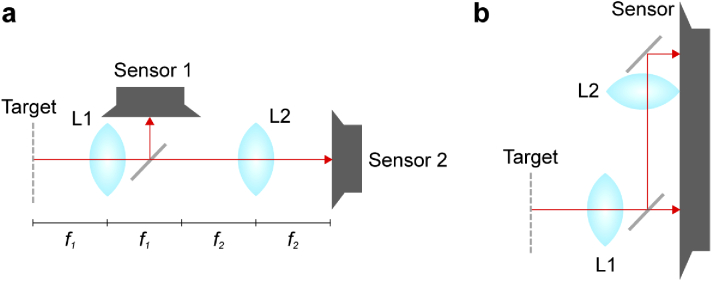
Schematic diagrams of the 4f architecture, where the red line indicates the optical path and L1, L2 are the system’s two lenses. a) A simple configuration with the system focal lengths $f_{1}$ and $f_{2}$ indicated. b) An alternative configuration with an additional folding mirror and a single larger sensor.

Phase imaging can be performed using a simple architecture based on a 4f correlator, shown schematically in Fig. 1. This is intuitive to consider because it can be simulated in terms of Fraunhofer propagation when all components are perfectly aligned [27]. While the size of the system is determined by the focal lengths $f_{1}$ and $f_{2}$, these lengths can be short provided the target can be close to the first lens. Further, as shown in Fig. 1(b)), it is possible to fold the system to work with dimensional constraints.

Beam propagation simulations were developed in MATLAB, using complex 2D arrays to represent input light fields at the object plane. Three input fields that were investigated; a small circular beam representing a pinhole at the object plane, a phase-only representation of the number 5 and a phase-only crest. The pinhole field enables an analytical check of the simulation results, while the phase-only number 5 provides a test of a non-centrally-symmetric target and simulates the birefringent target measurements described in Section 2.1.3. The crest was used as an additional multi-phase target for further testing.

Fraunhofer propagation was simulated using discrete 2D Fourier transforms to determine the intensity and phase distributions at the Fourier (2f) and image (4f) planes. As 2D Fourier transforms do not typically preserve the physical size of their array elements, it was essential to explicitly consider scaling to ensure that the simulations matched the expected sensor intensity distributions. This was achieved using the following scaling relationship [28]:

(1) $$L_{2f}=\frac{\lambda f}{\Delta L_{4f}}\;and\;\Delta L_{2f}=\frac{\lambda f}{L_{4f}}.$$

where *f* is focal length, $L_{2f,4f}$ is array side length at a given multiple of the focal length and *$\Delta L_{2f,4f}$* is array element size at that position. For simplicity, the two focal lengths in the simulated system were set equal to each other and to the experimental arrangement described in Section 2.1.3. The side length and element size for the 4f plane array were set to match the dimensions of the actual sensor. Consequently, it was necessary to downsample the 2f plane to determine the expected intensity distribution on the 2f sensor.

#### 2.1.2 Simulation: identifying and compensating for axial misalignment

Introducing axial misalignment places the system in the Fresnel regime, where beam propagation along the z axis is described by the paraxial diffraction integral. There are different numerical approximations for calculating this integral, depending on a critical distance parameter

(2) $$z_{crit}=\frac{N\Delta x^{2}}{\lambda }$$

where N is the number of array elements per side (where the array is assumed to be square), Δx is the size of the array elements and λ is the wavelength of the illuminating light. For distances shorter than $z_{crit}$, a convolutional approach may be taken that multiplies the field by the Fresnel kernel in the Fourier domain. For distances longer than $z_{crit}$, an approach involving multiplication by quadratic phase factors may be used; as the distance tends to infinity, this approach reduces to the Fraunhofer approximation [27]. These approaches were implemented in our simulation, then extended to describe 2D arrays and to include the backwards propagation required by the modified phase retrieval algorithms described below.

Axial sensor misalignment was introduced into the propagation simulations by adding steps of Fresnel propagation. Phase retrieval using the Gerchberg-Saxton algorithm was then attempted with no information about this misalignment. The deterioration of the phase retrieval results was assessed visually and using a mean-squared error (MSE) metric to compare with the known phase profile:

(3) $$MSE=\frac{\sum_{x}\sum_{y}\|E{\left(x,y\right)}_{recovered}-E{\left(x,y\right)}_{ideal}{\|}^{2}}{\sum_{x}\sum_{y}1}$$

where $E{\left(x,y\right)}_{recovered}$ is the recovered complex field and $E{\left(x,y\right)}_{ideal}$ is the expected ideal complex field.

An approach to correcting for axial sensor misalignment in post-processing was developed and tested. First, the degree of misalignment is determined using a known input target field, $E_{in}$ (e.g. a pinhole). A first Fresnel propagation correction is applied to this input using an initial guess of the distance parameter $dz_{1}^{\prime }$. The amplitude is then replaced with that measured on the 4f sensor, and the result propagated to the 2f plane via a Fourier transform. A second Fresnel propagation correction is applied using an initial guess of the distance parameter $dz_{2}^{\prime }$. The amplitude is then replaced with that measured on the 2f sensor, and the result is inverse propagated back to the 4f plane via an Inverse Fourier Transform, giving the output field $E_{out}$. The error between $E_{in}$ and $E_{out}$ is computed giving an error metric, ϵ, as:

(4) $$\epsilon =\sum_{x,y}\|E_{in}-E_{out}{\|}^{2}$$

ϵ is a minimum when the correct offset distances, $dz_{1}$ and $dz_{2}$ are used. A golden section search minimisation approach was applied using the MATLAB fminbnd function to determine the optimal values for $dz_{1}$ and $dz_{2}$. If only one sensor is displaced, the other dz value is fixed at zero. For a specific system with fixed misalignments, these values can be determined empirically prior to the first operation as a calibration step. This enables the phase retrieval algorithm to be modified to accommodate this additional distance and produce more accurate phase images.

**Fig. 2.**
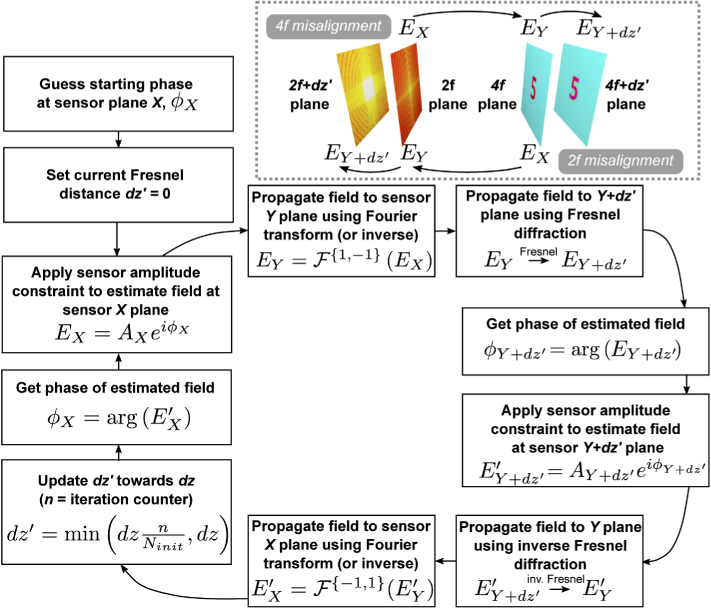
Algorithm showing recovery process for the case when the 2f or 4f sensor is misaligned in the z-axis, i.e. the 2f or 4f images are out of focus. Due to the challenges in algorithm convergence using Fresnel propagation, the Fresnel distance, $dz^{\prime }$ is slowly increased from 0 (i.e. the Gerchberg-Saxton case) to its true value, dz, which is determined in advance through a calibration process using a pinhole. A represents amplitude, ϕ represents phase, $E=Ae^{i\phi }$ represents a field and subscripts X and Y denote either the 2f or 4f plane as per the inset.

Second, once the degree of misalignment is known, a modified phase retrieval algorithm was implemented to reconstruct phase images, shown schematically in Fig. 2. It is based on the Gerchberg-Saxton algorithm with an additional Fresnel propagation step representing the misalignment distance dz. In general, iterative Fresnel propagation algorithms are known to suffer from stagnation if using fewer than three intensity planes [29]. However, as the axial misalignment becomes small, we expect the measured intensity to approximate the actual 2f or 4f plane. To reduce the likelihood of algorithm stagnation, we begin phase retrieval by setting the Fresnel distance $dz^{\prime }=0$, then slowly and linearly increase $dz^{\prime }$ up to the true value of dz. This approach provides a good initial approximation of the phase by exploiting the convergence properties of the Gerchberg-Saxton algorithm, then corrects for small differences by gradually introducing Fresnel propagation. By enforcing the measured intensity at two separate planes, this architecture avoids the twin-image problem [30,31].

#### 2.1.3 Experimental arrangement: 4f architecture

The presented 4f architecture is worth considering in the context of capsule endoscopy because it can be constructed from off-the-shelf components and provides a true optical image of the object plane in addition to a phase image. Comparing experimental and simulated phase imaging results provides an intuitive test of the analytical framework at the foundation of both architecture simulations. As such, a 4f-based phase imaging system was constructed for preliminary experimental measurements.

**Fig. 3.**
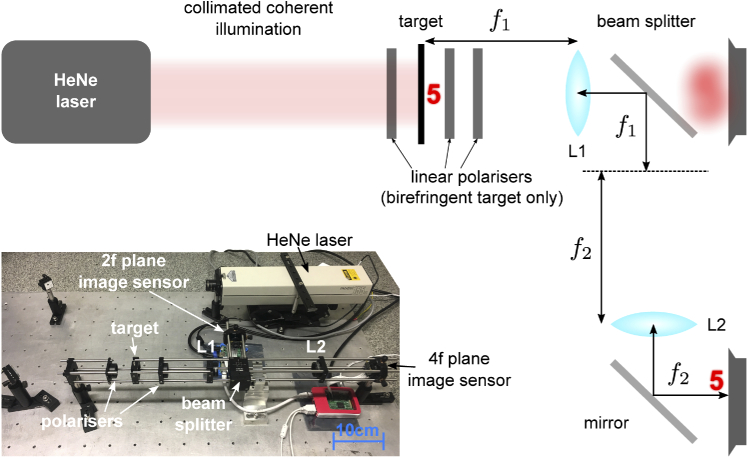
4f architecture: Schematic diagram and photograph of experimental arrangement

Phase imaging experiments were carried out using the apparatus shown in Fig. 3. All lenses had a focal length f = 100 mm (Thorlabs AC254-100-A-ML) to provide sufficient space for manual optical alignment and visual inspection throughout the beam path. The object plane was back-illuminated using a collimated 632.8 nm HeNe laser (SpectraPhysics 155SL) which was attenuated from its maximum power of 0.9 5mW to below 100 μW to prevent saturation at the sensor planes. A 50:50 non-polarising beam splitter (Thorlabs CM1-BS013) was used to sample the optical field at the 2f and 4f positions, where sensors were mounted on 3-axis translatable cage plates (Thorlabs CXYZ05/M) to facilitate alignment. All lenses and beam splitters had anti-reflection coatings appropriate for the incident wavelength; there were no discernible reflection artefacts.

Intensities at the 2f and 4f planes were recorded using Raspberry Pi v2.0 NoIR camera modules with their lenses removed. These modules contain Sony IMX219 8-megapixel sensors with 1.12 μm pixels. Data acquisition with both sensors was controlled from a single Raspberry Pi computer using an http server arrangement, and Python’s pycamera module was used to control acquisition parameters. The raw 10-bit values associated with the red component of the sensor’s Bayer filter were extracted and demosaiced in order to access the maximum available dynamic range and avoid the nonlinearities introduced by on-board gamma correction.

Measurements were made with two object plane configurations. Firstly, a 100 μm pinhole was used to acquire data that provides a direct comparison to the simulations. Secondly, a configuration comprising a birefringent resolution target (Thorlabs R2L2S1B) and three linear polarisers was used to produce features with re-configurable intensity and phase properties at the image plane. By positioning the polarisers at specific angles, characters on the target could be rendered invisible in intensity while retaining contrast in phase. A propagation model incorporating each of these elements was used to guide the rotation of linear polarisers to produce intensity-visible characters for initial setup and then move to a phase-only configuration.

Lateral sensor misalignment in a 4f correlator architecture can be identified experimentally by observing the image of a pinhole with the 4f sensor and observing a collimated beam with the 2f sensor. This facilitates initial alignment and quality control procedures during instrument manufacturing. Any remaining offset that is deemed to be acceptable can be accounted for in image post-processing by identifying the optical centre of each sensor using the same two inputs and symmetrically cropping both sensor arrays to accommodate the largest offset. This technique was applied in the experimental measurements conducted with this arrangement.

The size of the experimental arrangement was determined by the relatively long focal length (f = 100 mm) and simple optical path (see Fig 3 inset). The schematic diagram in Fig 1 shows a folded configuration that results in a different overall footprint and places both sensors in the same plane. The focal length was selected to allow polarising elements to be easily inserted around the object plane, and to enable direct observation of the optical field between components for troubleshooting purposes. The focal length can be significantly reduced without altering the fundamental properties of the system; indeed, the scaling relationship in Eq. (1) sets the 2f and 4f planes equal to each other for the purposes of Fourier transforms at a focal length of 5 mm. This configuration could therefore be plausibly implemented in a sufficiently compact way to be applied to capsule endoscopy.

### 2.2 Phase plate architecture

#### 2.2.1 Simulation: phase imaging

**Fig. 4.**
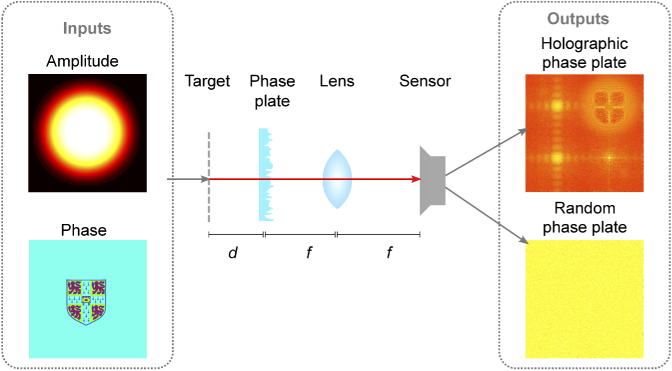
A phase imaging system based on a phase plate architecture. The input field first propagates a small distance, d, then is modulated by a phase plate. A 2f imaging system is used to form the Fourier transform of the modulated image on the sensor. Simulated intensity images are shown for two phase plate designs: a random design with pixels introducing a uniformly distributed phase-shift between 0 and 2π, and a design made up of the superposition of four holograms (see Fig. 5)

**Fig. 5.**
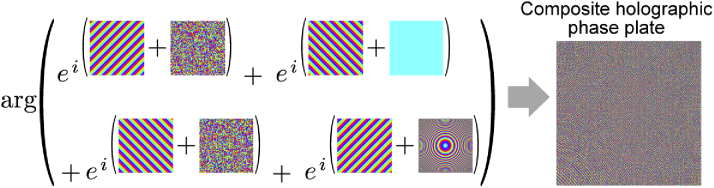
Construction of the holographic phase plate: Four different holograms (two random, one blank and one Fresnel lens) are each offset by a blazed grating and then a phase-only superposition is taken to form the final composite phase plate design.

A second possible architecture for low-cost, compact quantitative phase imaging uses a phase plate that scatters light in a controlled way before it is incident on the image sensor. A lens is used to Fourier transform the image after the phase plate before being incident on the sensor (Fig. 4). The phase plate can be designed in many ways; here we consider two approaches. The first is to use a purely random grating. This makes for simpler fabrication but it is not immediately possible to interpret raw sensor images, which is a disadvantage when trying to put a device in a specific position based on sensor feedback. A second design is proposed which overcomes this disadvantage with a superposition of four holograms: two different randomised holograms, a flat plane and a Fresnel lens (Fig. 5). The two random holograms scramble information, the flat phase produces a Fourier spectrum of the object and the Fresnel lens produces an approximate image of the object plane. A blazed grating is applied to each of these holograms to ensure their replay fields occupy different quadrants of the sensor (Fig. 5). This approach means that approximate images of the Fourier and object planes are recorded directly on the sensor, albeit at a reduced resolution and with some overlap between quadrants. Having this information available without processing enables practical placement and alignment of the system (e.g. navigating a capsule endoscope). The same reconstruction algorithm is used for both types of phase plate and requires only knowledge of the exact phase plate design and the distances between elements. Overlap of the quadrants does not affect reconstruction provided that these quantities are know.

In both cases, the recovery process relies on the phase plate mixing of phase and amplitude information in adjacent areas. By recovering an image with lower resolution than the sensor, the phase information can be extracted from the amplitude information. This is analogous to using two amplitude-only images to extract a phase profile in the conventional Gerchberg-Saxton algorithm. It also has parallels with Shack-Hartmann wavefront sensors and quadriwave lateral shearing interferometers, both of which sacrifice sensor pixels to determine optical phase. The presented approach permits higher utilisation of sensor pixels because the Fourier transform typically has a smaller support than the real space image and so the four Fourier space images experience minimal overlap on the sensor. [20].

**Fig. 6.**
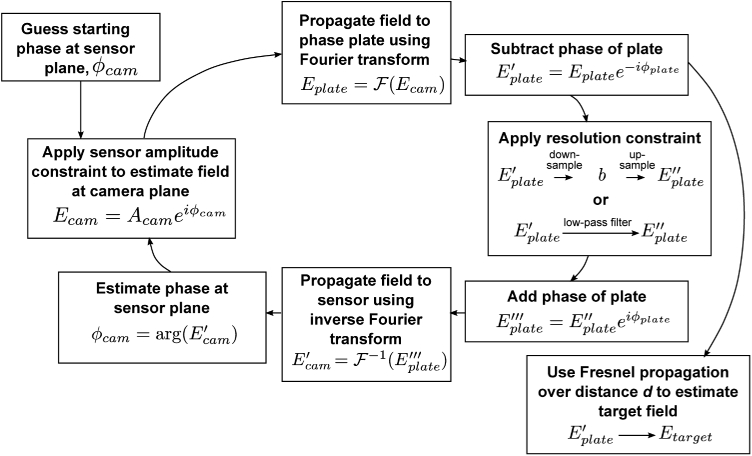
Algorithm detailing how phase images are recovered from sensor intensity images using prior knowledge of the phase plate design. A represents amplitude, ϕ represents phase, $E=Ae^{i\phi }$ represents a field.

The recovery algorithm is a modified version of that presented in [32], which is in turn inspired by the Gerchberg-Saxton algorithm: An amplitude constraint is applied in the Fourier domain of the phase plate (the sensor), and a resolution constraint is applied in the phase-plate domain, after correcting for the phase plate (Fig. 6). This modification is similar to the finite support constraint commonly used in the hybrid input-ouput algorithm [15] except the finite support is in the Fourier plane. The use of this additional constraint in the Fourier domain appears to limit the twin image problem, which we do not observe, in line with previous approaches [19,31].

In our initial implementation, one iteration of the algorithm implemented in MATLAB takes about 300 ms, but this could be reduced through code optimisation, re-implementing in C++ or utilising GPU processing.

#### 2.2.2 Simulation: evaluating manufacturing and alignment constraints

The profile of the phase plate must be known in advance for the algorithm to work. This could be achieved simply by using the fabrication design as a direct input to the algorithm, which would work within an error tolerance as established in Section 3.3, or by imaging the fabricated phase plate thickness using, for example, an ellipsometer. To improve manufacturability we next consider variations to the phase plate design and the effect of imperfect characterisation. First, we increase the level of quantisation of the phase plate, i.e. with pixels larger than the camera pixels, to make manufacturing the phase plate easier. This is achieved by first designing a phase plate and then averaging groups of 2×2, 4×4 or 8×8 pixels. Next, we consider the impact of noise arising from fabrication or characterisation error. This is tested by adding random phase noise to the simulated phase plate and using it to recover images from data generated using the ideal plate. Finally, we consider physical misalignment of the plate, specifically lateral translation by up to 13 % of lens focal length and axial translation of both the grating (up to 10 % of lens focal length) and the sensor (up to 50 % of lens focal length), by introducing an offset into the simulation. This is larger than the anticipated misalignment in the construction of such a system.

## 3. Results

### 3.1 4f architecture: comparison of simulated and experimental phase images

**Fig. 7.**
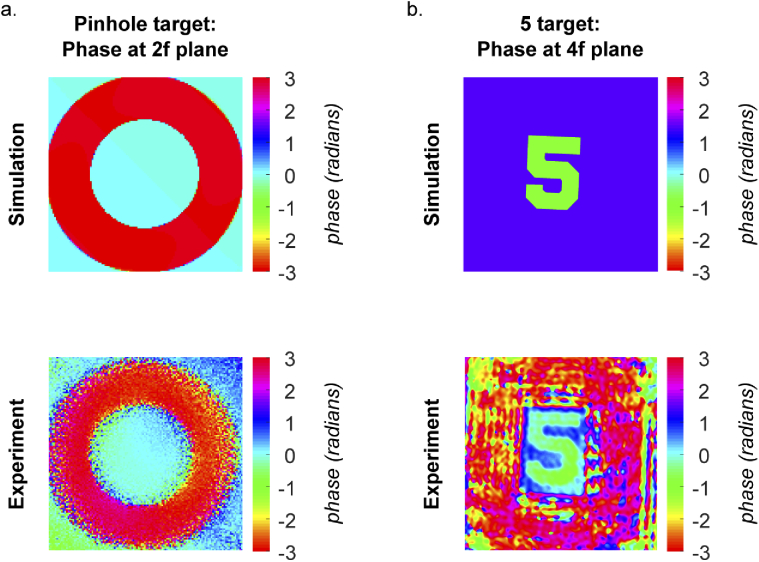
Simulated and experimentally measured phase images produced with a 4f system architecture: a) pinhole target, b) birefringent target

Phase imaging experiments were conducted using the experimental arrangement described in Section 2.1.3. A pinhole was selected as the first target because the Fourier transform can be determined analytically to be a 2D sinc function. This enabled the simulated result to be independently verified prior to comparison with the experimental result. A 100 μm pinhole was selected to produce a clear, distinctive phase profile at the 2f plane for comparison with experimental results. With the pinhole illuminated, the intensity on both sensors was recorded and phase retrieval performed to produce a phase image. Figure 7(a) shows this experimentally-acquired phase image alongside the simulated equivalent, demonstrating good agreement between the two and showing that Raspberry Pi image sensors are sufficient for conducting phase imaging experiments.

A birefringent resolution target was then measured to test the system’s ability to produce images when the amplitude contrast is low and the phase contrast is high. Again, the intensity was recorded on both sensors and the experimental phase retrieval result was compared with the simulation results for the same system. Figure 7(b) shows that the experimentally-measured phase contrast is in good agreement with the simulated result, which was based on the known difference in birefringence between the two regions.

The phase images shown in Fig. 7 demonstrate the feasibility of the 4f correlator approach for widefield phase imaging and show that the simulations have been successfully implemented with the correct scaling so as to faithfully represent the physical phase imaging results.

### 3.2 4f architecture: axial alignment tolerance and correction simulations

**Fig. 8.**
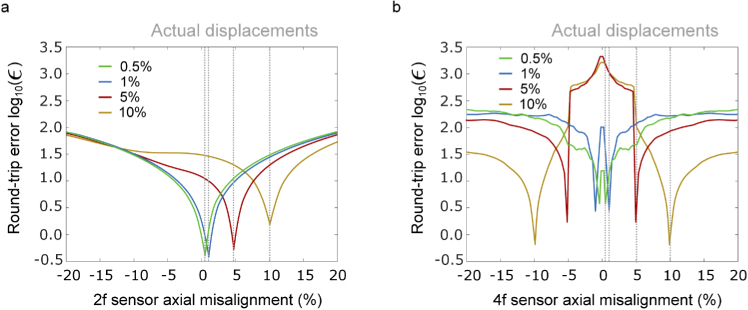
Identification of axial misalignment (dz) through minimisation of round-trip error for a) dz (2f sensor) = 0.5 %, 1 %, 5 %, 10 %, and b) dz (4f sensor) = 0.5 %, 1 %, 5 % and 1 0%. ϵ represents the round-trip error defined in Eq. (4), percentages are relative to focal length.

The purpose of this investigation was to determine the extent to which axial sensor misalignment affects phase imaging performance, and whether this can be corrected for in-situ. Using the axial misalignment calibration method described in Section 2.1.2, the round-trip error was observed to show a clear minimum at the correct axial misalignment for a range of values, demonstrating that this can be determined from a calibration step. Figure 8 therefore shows the successful implementation of the axial misalignment calibration method. Propagation of the pinhole target through the 4f system was simulated with +0.5 %+ 1.0 % +5 % and +10 %(given as a percentages of lens focal length to ensure scale invariance) misalignment of the 2f and 4f sensors individually, then the calibration algorithm was used to independently identify the misalignment. There is degeneracy in the 4f case because the +dz case differs from the -dz case by a parabolic phase mask of the same magnitude but different sign. The different signs of dz cancel this difference, so the two cases are degenerate. This results in a global phase error, which will not reduce contrast for imaging phase as it is held in the relative phase between pixels. These results show that it is possible to identify the extent of axial sensor misalignment in-situ.

**Fig. 9.**
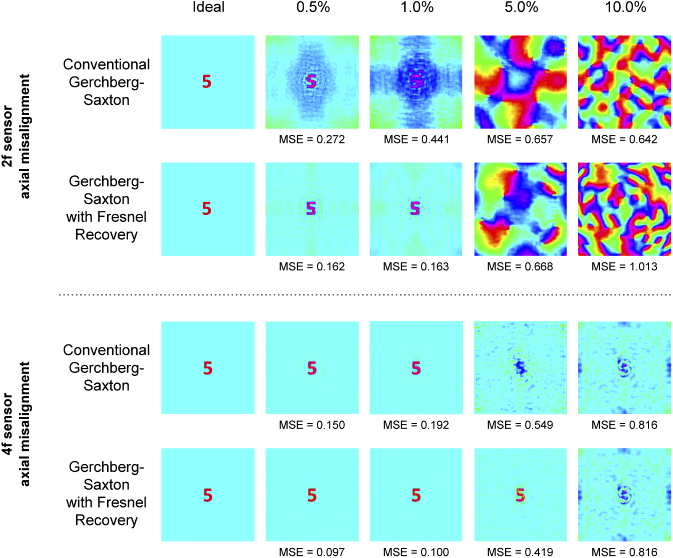
Plot showing recovery of simulated phase images using the 4f correlation system of Fig. 1 with different simulated displacements of the 2f and 4f planes, and phase retrieval results with and without correction for axial misalignment

Figure 9 shows the impact of axial misalignment correction on simulated phase imaging results. In each set, the top row shows phase retrieval performed assuming a perfect 4f system, and the bottom row shows phase retrieval using the algorithm described in Fig. 2. In the 4f misalignment case,the Gerchberg-Saxton algorithm recovers phase accurately for displacements of up to 1 % (MSE < 0.2). At 5 % misalignment, the modified algorithm of Fig. 2 improves phase recovery (MSE = 0.42). These results show that misalignment of the 2f sensor has a significant impact of phase retrieval, and that retrieval quality is improved by applying a misalignment correction in both the 2f and 4f cases.

**Fig. 10.**
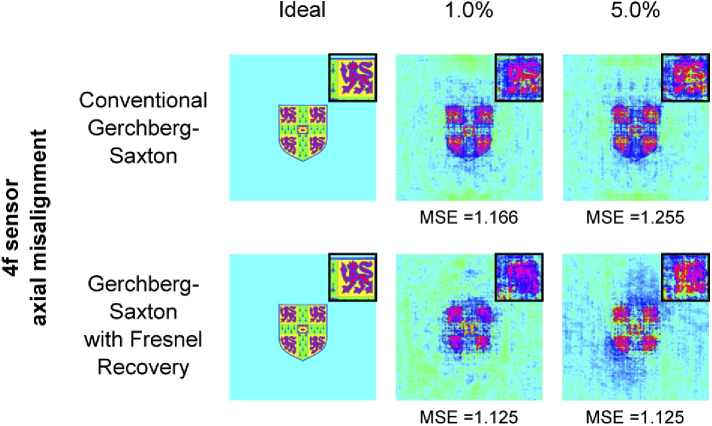
Simulated imaging of a multi-level phase target at 1% and 5% axial misalignment of the 4f plane. Broad recovery of the original input is possible, though with reduced MSE.

Finally, Fig. 10 shows simulated phase imaging with the 4f system using a more complex multi-level phase target: the crest used to evaluate phase-plate performance. Though the recovered phase is recognisable, including multi-level phase features such as the lion outlines, the quality reduction is indicative of this architecture’s sensitivity to axial misalignment. Some improvement in MSE is obtained through use of the modified recovery algorithm.

### 3.3 Phase plate architecture: simulation of manufacturing and alignment constraints

**Fig. 11.**
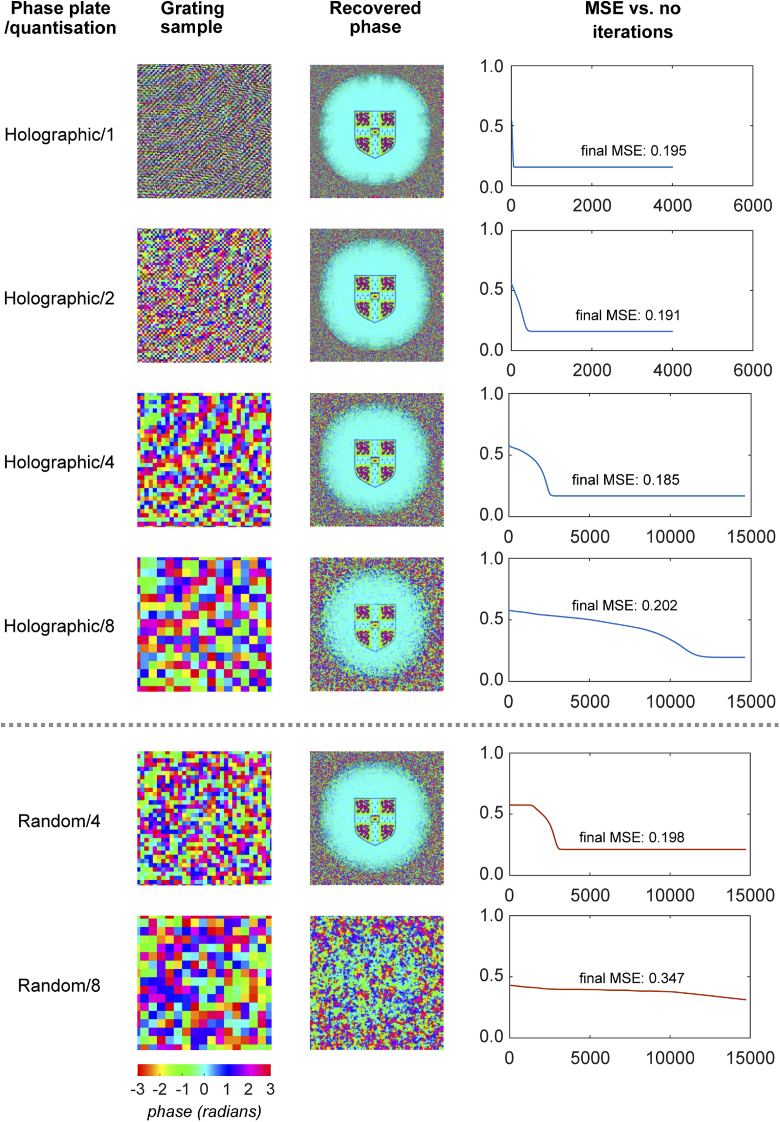
Impact of phase plate quantisation on simulated phase retrieval performance, showing a sample area of each grating, a recovered image and a plot of MSE vs. iteration during the recovery algorithm.

The first aspect of the phase plate architecture that was investigated was the impact of introducing quantisation. This has the benefit of making phase plates easier to manufacture and characterise, provided that it does not significantly reduce phase imaging performance. Figure 11 shows the simulated recovered phase and error curve as a function of the number of algorithm iterations for holographic and random gratings with different levels of quantisation. For the holographic grating, an 8-fold increase in phase plate ‘pixel’ size increases the number of required iterations, but does not significantly impact upon the MSE of the phase imaging result. For the random grating, a 4-fold increase in pixel size was the maximum that could be accommodated while maintaining the ability to recover phase. However, it is noted that convergence time is increased by a factor of ∼100 for the 8-fold quantisation of the phase plate. Lower quantisation will result in a more disordered field captured on the sensor due to increased scattering, comparable to transmission through a rougher surface. We speculate that when using iterative recovery algorithms, this more abrupt change results in larger moves around the candidate solution space per iteration, which may explain the reduction in convergence time. In any case, there is a clear trade-off between phase plate feature size and algorithm convergence time. In the current MATLAB implementation each iteration takes ∼300 ms. For the holographic grating with no quantisation, convergence requires ∼ 20 iterations, or about 6 seconds, though this speed could be reduced significantly by further code optimisation. In the worst-case scenarios, however, convergence could take many hours.

**Fig. 12.**
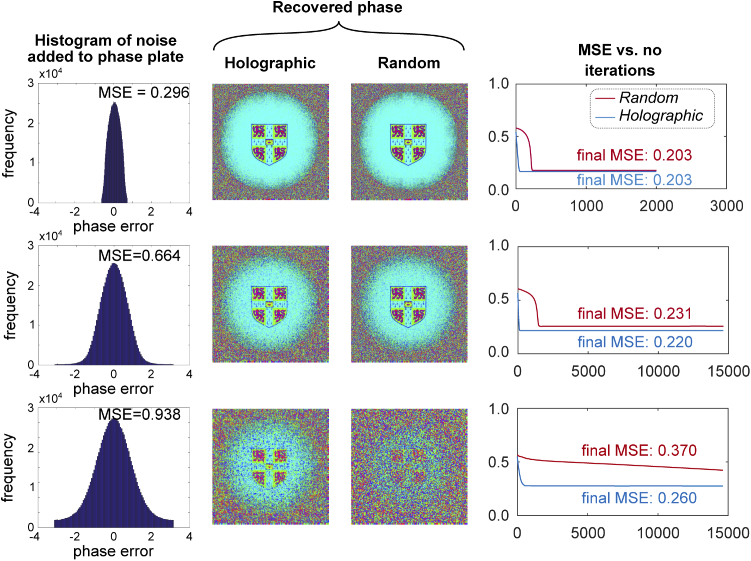
Impact of phase plate noise on simulated phase retrieval performance

Next, different levels of noise were introduced into the simulated phase plate to represent the potential impact of manufacturing quality on phase retrieval. Noise levels were selected so as to explore the limit where accurate reconstruction was no longer possible. Intuitively, if phase were totally randomised by noise then reconstruction would not be possible so we wish to find a point in between where reconstruction is still possible and computationally tractable, and translate this to a physical manufacturing tolerance. Figure 12 shows the results of these simulations; while both phase plates can accommodate the lowest level of noise, performance deteriorates more rapidly for the random phase plate. We note that acceptable performance is still obtained by the case with phase-plate noise MSE of 0.664. This corresponds to a standard deviation of ±1 radian in fabricating the phase plate, which for a plate designed from glass to be used in air would correspond to a thickness tolerance of ± 0.2 μm. This is well within the limits achievable using lithography which are typically of the order of a few nm [33]. Further, it is noted that convergence time is not significantly altered due to the presence of noise.

**Fig. 13.**
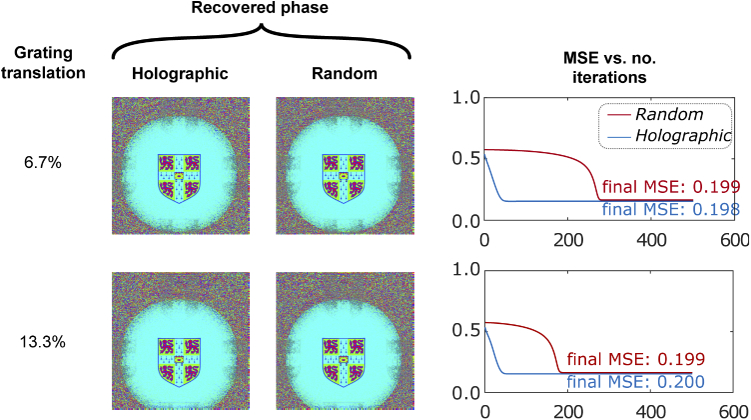
Impact of phase plate translation on simulated phase retrieval performance

The effect of lateral translation was then examined within the simulation framework by translating the phase plate element and performing phase retrieval with the same crest input field (Fig. 13). The recorded intensity images on the sensor are unaffected because translations correspond to linear phase tilts in the Fourier domain. Therefore, there is no reduction in MSE when recovering images using the phase plate (MSE = 0.200). However, because an incorrectly translated version of the phase plate is used during the phase compensation step, the result is that that reconstructed object appears to be translated. This is easily corrected, for example by cropping and translating recovered images.

From the above error curves it is observed that the designed holographic phase plate produces significantly faster convergence than the random phase plate. We speculate that this is because the designed phase plate tends to focus power in certain regions of the sensor, meaning that the phase of pixels in these regions has a greater impact than those in regions with little power. Therefore, only a relatively minor change to the estimated phase can produce a large reduction in error, enabling a quick reduction in error in early iterations. By contrast, the random phase plate spreads power more broadly over the sensor area so minor changes to pixels early in the algorithm reduce the error by relatively small amounts.

**Fig. 14.**
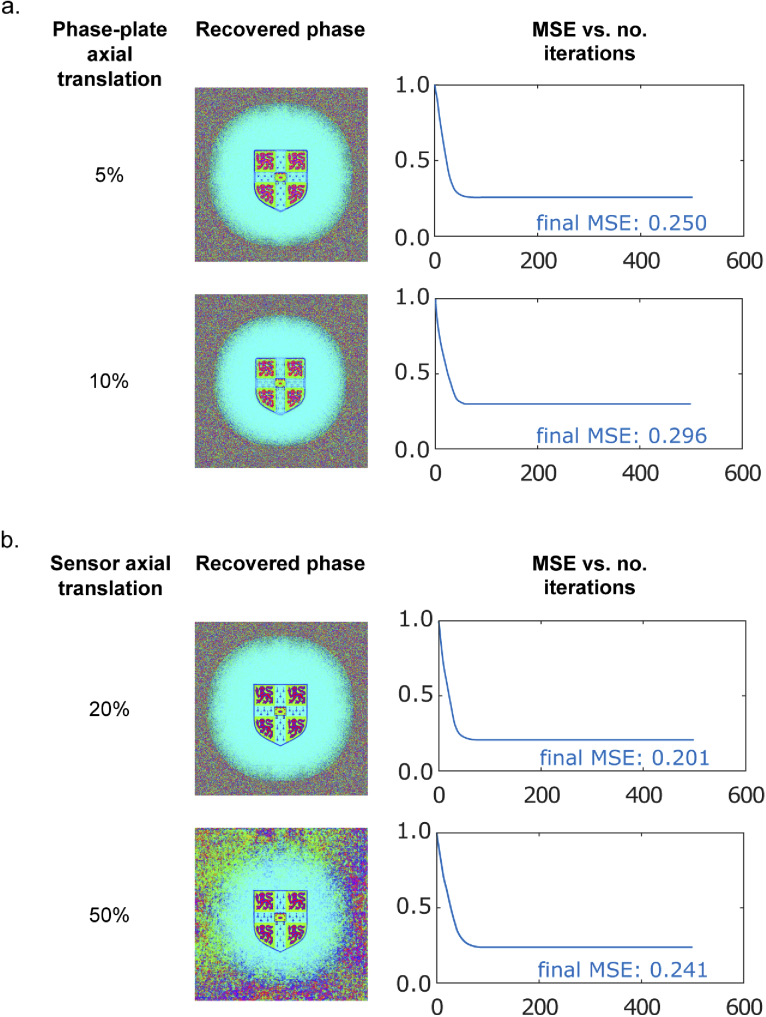
Impact of phase plate axial translation on simulated phase retrieval performance

Finally, the effect of axial misalignment was tested by performing phase retrieval after introducing this into the simulations (Fig. 14). The phase-plate was translated by 5% then 10% which resulted in an increase of MSE to 0.250 and 0.296, respectively (Fig. 14(a)). This arises from defocus error so could be corrected by additional Fresnel propagation steps. However, the visual quality of recovery is high and so it may be concluded that 5-10% misalignment is permissible. The sensor permits as much as 50% axial misalignment error while still producing small MSE values and visually accurate results (Fig. 14(b)). This is likely due to the nature of the recovery algorithm (Fig. 6) in which the sensor field estimate is propagated to the phase plate via the lens, where a resolution constraint is then applied. Then, the reverse of this propagation is used to produce the next estimate of the field at the sensor. Even if this propagation does not match the physical propagation due to misalignment, the algorithm remains robust because the actual propagation used by the algorithm is always exactly reversed. This is a key benefit of approaches that measure only a single propagation plane as it removes one source of error – the relative distance between planes. However, the resolution constraint is therefore applied in a defocussed plane, which may account for the small increase in MSE. Overall, it is evident that the phase-plate architecture is relatively robust to axial misalignment.

## 4. Discussion

Phase imaging in endoscopy could aid clinical decision making by enhancing contrast for early stage disease in vivo [2–4]. Recent work on low-cost solutions for quantitative phase imaging has targeted microscopy applications, producing valuable results that address a different set of constraints [34,35]. To utilise phase imaging in a capsule endoscope, it must be feasible to fit key components within a capsule (typically 11 mm × 25 mm) in a way that is relatively low-cost and robust to manufacturing variations. The approaches presented here have the potential to work within these constraints and may also find application in open-source hardware projects for low-resource settings [36,37]. The possibility of implementing these technique with low-coherence sources such as diode lasers means that the illumination source could feasibly be housed inside the capsule as opposed to remotely, as is the case for OCT-based capsule imaging [38]. We consider modifications to the detection path of an imaging system, though modifications to the illumination path could be considered for alternative methods of measuring scattering (e.g. structured illumination) [39].

We have investigated two non-interferometric phase imaging architectures. The first, a 4f correlator architecture, was investigated in simulation and experimentally. We showed, through simulating the imaging of a known pinhole target, that axial sensor misalignments can be characterised via an optimisation algorithm. Further, using a modified phase retrieval algorithm incorporating a Fresnel propagation correction step, this characterised axial misalignment can be used to enable phase imaging with a 2f sensor axial misalignment of up to 1% and a 4f axial misalignment of up to 5%. The system appears to be more tolerant to misalignment of the 4f plane (i.e. the object plane). This is likely because most of the information required for reconstruction is contained in the 2f plane (the Fourier plane) – in many cases it is possible to reconstruct phase images from this plane alone [15]. Finally, we show experimentally that using this design, quantitative phase images can be recovered using two Raspberry Pi cameras and light delivered via an optical fibre, demonstrating the feasibility of the approach. Using pinhole and birefringent targets we demonstrate good agreement between simulation and experiment. Further, the HeNe could be replaced with a low-cost diode laser that could fit inside a small form-factor instrument, and although two sensors are used here, it would be possible to use one sensor with two separate regions. We note that the Fourier-transform relationship between the 2f and 4f sensors makes correct scaling crucial for retrieving phase from the recorded intensity distributions. For a wavelength of 632.8 nm and a 2464×2464 array of 1.12 μm pixels, a focal length of 4.9 mm results in Fourier plane array elements of equal size to those in at the image plane. This means that data can be acquired with sensors with the same sized pixels (or parts of the same sensor) without requiring that the Fourier plane be downsampled, which could result in information loss at high spatial frequencies. This indicates that scaling a system down to the dimensions of a capsule endoscopy (approximately 11 mm x 25 mm) would be possible and not detrimental to phase imaging performance.

The 4f architecture is not without its limitations. The resolution is limited by two physical constraints: the size of the sensor in 2f plane, which limits the maximum spatial frequency that can be constructed, and the pixel size in the 4f plane. The first constraint is adjusted by changing the size of the first lens, such that the desired spatial frequencies are recorded. Alternatively, a larger sensor may be used but this may be impractical under capsule size constraints. The second constraint is modified by changing the focal length of the second lens to magnify or de-magnify such that the smallest features are matched to the pixel pitch, which is typically 2-10 μm. Similarly, the field of view is limited by the pixel size in the 2f plane and the sensor size in the 4f plane. There is therefore a trade-off between field of view and resolution in selecting lenses. The appropriate balance will be application dependent: for example, microscopic analysis of tissue requires higher resolution, while wide-field screening of large samples needs a larger field of view. The algorithms could be improved to work for a wider range of axial misalignment. Due to the known poor convergence properties of iterative Fresnel propagation, this design may benefit from the use of alternative recovery approaches, such as the transport of intensity equations. These provide analytical approximations to phase; while they are not robust to noise [29], they could form robust starting points for a Fresnel iterative approach. In the context of the current results, the 1 % sensor misalignment tolerance demonstrated in simulation is a reasonable requirement for practical low-cost construction. With a 10mm focal length lens this would corresponds to a 0.1 mm tolerance, which is within the range of commercial 3D printers.

The second design, based on a phase plate, was investigated in simulation. The replacement of one lens with a phase plate removes one distance constraint, increasing compatibility with the space constraints of a capsule endoscope. Two phase plate designs were presented: a holographic design comprising a superposition of four holograms, and a random design in which each element is selected randomly to have a phase shift between 0 and 2π. We present an iterative recovery algorithm that, with knowledge of the phase plate, is able to reconstruct phase based on a single intensity measurement at the expense of resolution. Initially, the phase plate applies a different phase at each effective pixel of the input field, whose size is determined by the finite extent of the sensor located in the Fourier plane. In this case, our algorithm convergences within ∼20 iteration, corresponding to around 6s. With further code optimisations, e.g. using C/C++ instead of MATLAB and exploiting high-speed GPUs, we believe it should be feasible to reduce this to <1 s, approaching real-time performance. We next show that without needing to modify the recovery process the pixels on the phase plate can be as much as 8 times this size, significantly reducing the requirements for precision manufacturing and hence cost. This comes at the expense of convergence time during recovery, with the holographic plate producing convergence  100 fold slower, and the random phase plate not converging at all within our limit of 15000 iterations. This corresponds to recovery times of several minutes to several hours, making real-time operation more challenging. Phase plates with smaller features are therefore preferable. A further consideration for manufacturing is the requirement for precision in the phase changes introduced at each pixel. We show that a mean-squared error in phase plate fabrication of 0.664 still enables accurate phase recovery. This corresponds to an error of ±1 radian at each pixel, which for a glass phase plate in air corresponds to a thickness tolerance of ∼ 0.2 μm, comfortably achievable using standard lithography processes [33]. Unlike quantisation effects, this does not significantly reduce recovery time, which is promising for making practical devices. Finally, we show that a transverse misalignment of the phase plate of up to 13 % does not significantly degrade phase recovery or increase recovery time, resulting simply in a corresponding translation of the recovered phase image.

The phase-plate approach also has associated limitations. The first is fabricating the phase plate: though this can be done using lithography techniques followed by etching (e.g. using glass a maximum etch depth of ∼1.4μm would be required at 650nm), it is arguably a more complex approach than the 4f correlator design that exclusively uses standard components. The second is that because only one lens is used, there is less freedom to trade off resolution and field-of-view for a specific application. The scattering nature of the phase plate means that the raw sensor images may not resemble the sample. This may make real-time positioning and registration challenging, compared with the 4f-correlator design in which the 4f sensor records the amplitude at the sample. The holographic phase-plate design goes some way towards by providing an approximate image of the sample in one quadrant.

While it is not possible to put a specific price on a phase imaging capsule endoscope at this stage, consideration of the key cost drivers indicates that there are no fundamental obstacles to low-cost manufacturing at scale. The presented experimental results were produced with Raspberry Pi sensors which currently retail for £24 and contain commodity CMOS sensors that could be obtained in bulk at a lower price. Similarly, both systems could be implemented with lenses that could cost around £5 when manufactured in high volumes [40] and with laser diodes which can be purchased for less than £10 from standard component manufacturers. Our investigation of phase plate parameter and misalignment tolerances places manufacturing requirements within the realms of standard lithographic and commercial 3D printing techniques respectively. While the cost of producing a specific system depends on further definition of its specifications and requirements, neither architecture needs to contain any intrinsically expensive parts and both should be feasible to manufacture at scale at a low cost in the context of phase imaging microscopy.

To summarise, the presented 4f architecture is simpler than the phase plate architecture in that it can be constructed from standard components and provides a direct image of the object plane for reference. It is important that scaling is carefully considered in order to correctly relate the experimentally-measured 2f and 4f planes, and also to achieve the required balance of spatial resolution at 4f and spatial frequency resolution at 2f. Conversely, the phase plate architecture has fewer optical components overall and opens up a different range of phase retrieval algorithms. A phase plate with the presented holographic design performs better than a random phase plate and provides a reference image, albeit one that must be reconstructed algorithmically.

## 5. Conclusion

Our results indicate that it is feasible to produce a low-cost, compact phase imaging system using two different system architectures; one based on a folded 4f correlator, the other on a holographic phase plate. We have demonstrated, in simulation, a technique for determining and then correcting for axial sensor misalignment in situ, providing a feasible calibration step for the manufacture of phase imaging systems based on a modified 4f correlator. We then demonstrated in simulation a phase-plate architecture for phase imaging and presented an associated recovery algorithm that trades-off spatial resolution for phase recovery time. We showed that this algorithm reliably converges to the correct phase under a variety of conditions likely to be encountered in low-cost fabrication of the phase-plate: reduced spatial resolution, reduced depth (i.e. phase) resolution and transverse misalignment. Finally, we experimentally demonstrated phase imaging using the 4f design, showing that phase can be accurately recovered using low-cost Raspberry Pi sensors. We conclude that while the phase plate architecture is more robust to misalignment, both architectures merit further development with the goal of realising a low-cost, compact system for applying phase imaging in capsule endoscopy.

## Data Availability

Data for this publication is available at https://doi.org/10.17863/CAM.56831.
